# Necrotizing Sarcoid Granulomatosis Mimicking Lung Malignancy: MDCT, PET-CT and Pathologic Findings

**DOI:** 10.5812/iranjradiol.6572

**Published:** 2012-03-25

**Authors:** Hilal Sahin, Naim Ceylan, Selen Bayraktaroglu, Sezai Tasbakan, Ali Veral, Recep Savas

**Affiliations:** 1 Department of Radiology, Faculty of Medicine, Ege University, Izmir, Turkey; 2 Department of Pulmonology, Faculty of Medicine, Ege University, Izmir, Turkey; 3 Department of Pathology, Faculty of Medicine, Ege University, Izmir, Turkey

**Keywords:** Lung Neoplasms, Positron-Emission Tomography, Tomography, X-Ray Computed

## Abstract

Necrotizing sarcoid granulomatosis (NSG) is a rare disease which is classified in the spectrum of pulmonary angiitis and granulomatosis. It is a variant of sarcoidosis and differs from it histologically. Diagnosis is based on the pathological features, but radiology may help in the differential diagnosis. It is characterized by alveolar infiltrates or parenchymal nodules in multidetector computed tomography (MDCT). We report a case of a 50-year-old man with the diagnosis of NSG mimicking lung malignancy. Radiological and pathological findings and also the destructive course of the disease will be discussed.

## 1. Introduction

Necrotizing sarcoid granulomatosis (NSG) is a rare entity which was first described by Liebow in the spectrum of pulmonary angiitis and granulomatosis in 1973 [1]. Five distinct types of pulmonary angiitis and granulomatosis are recognized with similar radiological features [2]. NSG is one of these clinical syndromes which is described in angiocentric forms of granulomatosis [3]. It is usually diagnosed on the basis of pathological features and has some common clinical and histological findings with sarcoidosis [4]. Radiological findings are not specific. NSG cases usually present with multiple parenchymal nodules with or without cavitation. Solitary nodule or a mass is not a common manifestation in NSG [1]. Hereby, we describe a case of a 50-year-old man with a lung mass mimicking malignancy which was pathologically diagnosed as NSG. We also discuss the radiological and pathological findings regarding the atypical fulminant destructive course of the disease.

## 2. Case Presentation

**Figure 1:**
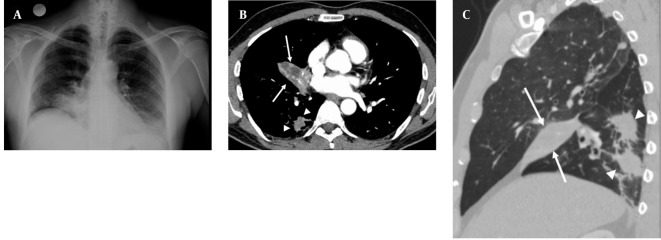
A 50-year-old man presenting with shortness of breath A, Posterior-anterior chest radiograph shows opacity in the right lower lung zone above the diaphragm deleting the right cardiac border; B, Axial CT image shows a mass (arrows) with hypodense areas in the middle lobe and irregular nodules in the right lower lobe (arrow heads); C, Sagittal reformatted CT image shows right middle lobe collapse caused by the mass.

**Figure 2:**
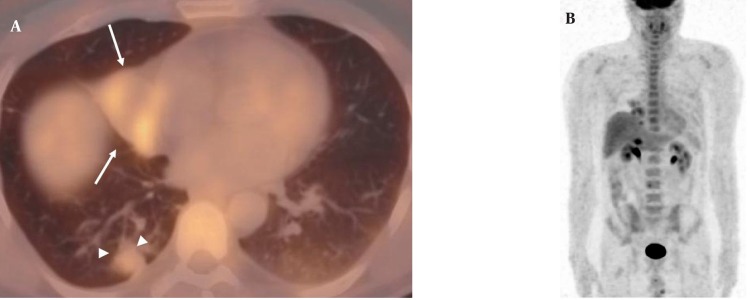
A, Axial PET-CT fusion image demonstrates that both the mass (arrows) and nodules (arrowheads) show FDG uptake with a SUVmax value of 2.9 and 2.5; B, Coronal MIP PET image shows no extrapulmonary involvement. (MIP: maximum intensity projection, SUVmax: maximum standardized uptake value, PET-CT: Positron emission tomography-computed tomography, FDG: Fluorodeoxyglucose)

**Figure 3:**
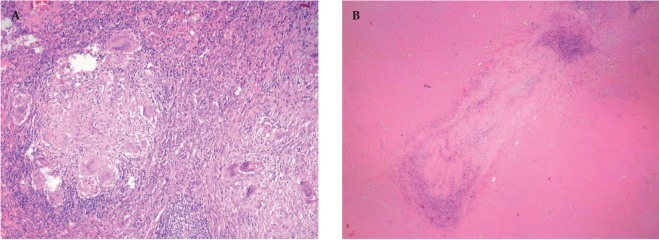
Histologic staining of the mass in the middle lobe A, Haematoxylin-eosin stain demonstrates coalescing sarcoid granulomas without necrosis (Haematoxylin-eosin × 200); B, Vasculitic involvement is seen between areas of large fibrinoid necrosis (Haematoxylin-eosin × 100).

A 50-year-old nonsmoking man presented with a 1-month history of cough and increasing shortness of breath in August 2007. He had no history of fever, night sweats, weight loss, chest pain, palpitation, arthralgia or skin rash. There was no history of allergy or systemic disease. On admission, he was afebrile with a respiratory rate of 22. The breath sounds were decreased in the rightlower zone. The rest of the physical examination was unremarkable. Chest radiograph on admission showed an ill-defined opacity in the right lower lung zone along the right cardiac border (Figure 1A). The laboratory tests including the complete blood count, biochemical and tumor markers were within normal limits. He underwent a contrast enhanced multidetector computed tomography (MDCT) examination of the thorax which showed a mass with a diameter of 3 cm near the major fissure in the middle lobe, narrowing the middle lobe bronchus (Figure 1B and C). The mass had sharp, smooth margins and a heterogeneous internal structure with hypodense areas in it. Accompanying the mass, there were peripheral nodular opacities with irregular margins in the right lower lobe. There was no lymphadenopathy or accompanying pleural effusion on MDCT. Bronchoalveolar lavage and transthoracic fine needle aspiration biopsy (FNAB) revealed benign cytology, but a malignant mass was suspected clinically and a positron emission tomography-computed tomography (PET-CT) scan was done for diagnosis and staging of the disease. On PET-CT, both the mass and nodules showed fluorodeoxyglucose (FDG) uptake with a maximum standardized uptake value (SUVmax) of 2.9 and 2.5, respectively (Figure 2A). There was no pathological FDG uptake in the mediastinal lymph nodes. Extrapulmonary involvement was not present (Figure 2B). Since malignancy could not be excluded by bronchoalveolar lavage, FNAB or PET-CT scan, percutaneous FNAB was repeated. The two consecutive biopsies revealed benign cytology and the patient only took symptomatic treatment. In October 2007, since the patient had progressive dyspnea and weight loss, a wedge resection of the mass and nodules was performed through a right thoracotomy. In histological staining, coalescing sarcoid granulomas and vasculitic involvement were seen between areas of large fibrinoid necrosis representing the diagnosis of NSG (Figure 3A and B). After that, steroid therapy was proposed and the patient’s complaints regressed.

**Figure 4:**
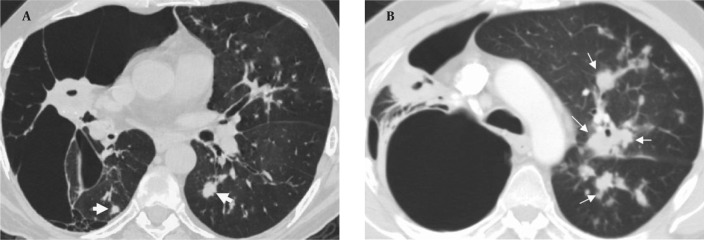
A, Axial CT image shows giant bullae in the right lung replacing normal parenchyma. Note the nodules in both lower lobes (arrows); B, Axial CT image reveals centrilobular acinar opacities (arrows) in the left upper and lower lobes which represent tuberculosis infection.

In September 2009, the patient was admitted to our hospital due to respiratory distress, cough and purulent sputum. Chest CT revealed giant bullae in the right lung with partial aeration of the right lower lobe (Figure 4A).

The bullae appeared during the follow-up and were considered as a sign of vanishing lung syndrome. On the other hand, some new centrilobular opacities in the left upper and lower lobes were recognized (Figure 4B). Acid fast bacilli consistent with Mycobacterium tuberculosis were present in the sputum and the diagnosis was confirmed in sputum cultures. Steroids were discontinued and the patient underwent antituberculous treatment.

## 3. Discussion

The spectrum of pulmonary angiitis and granulomatosis was first discussed by Liebow in 1973 and five distinct clinical syndromes were described: Wegener granulomatosis, lymphomatoid granulomatosis, NSG, bronchocentric granulomatosis and Churg-Strauss syndrome (allergic angiitis and granulomatosis) [1]. These entities are characterized by vasculitis, granulomatous inflammation and parenchymal necrosis in pathological examinations [5]. Diagnosis is essential since they may have a destructive course unlike pulmonary metastasis and infection which are considered in the differential diagnosis. NSG which is a rare systemic disease is at the extreme end of this spectrum. It is believed to be a variant of classical sarcoidosis, but the relation between them is an issue of debate. However, it differs from sarcoidosis histologically with more prominent vasculitis, marked necrosis and rare hilar lymphadenopathy [6]. The etiology and pathogenesis of the disease are unknown. It is usually seen in the late forties and is predominant in women [7]. Clinical presentation is variable and nonspecific. Symptoms may be absent or sometimes may mimic malignancy. When extrapulmonary involvement occurs, systemic manifestations are much more common [4].

Radiologic findings are not specific, but may sometimes give clues for differential diagnosis. Quaden et al. reported CT findings of NSG in 14 patients [4]. Alveolar infiltrates particularly common in the subpleural space were the most common features in this study. In addition, solitary or multiple nodules, in a range of 2-4 cm in diameter, without preference for any lobe were determined. Cavitation, hilar or mediastinal lymphadenopathy and pleural thickening were less common features. In our case, there were multiple irregular nodules as well as a well-defined mass with necrosis and no cavitation or pleural thickening accompanied these findings. Juxtalymphatic distribution of granulomas may mimic sarcoidosis, but the lower prevalence of mediastinal and hilar lymphadenopathy and the tendency of the nodules to cavitate may help distinguish NSG from sarcoidosis [8]. Rarely, NSG may present as a mass in the lung parenchyma, as in our case. Necrosis or cavitation may not allow distinction from malignancy; therefore, biopsy is required. FDG-PET has a value in the staging of known or suspected sarcoidosis, detecting occult diagnostic biopsy sites in patients with sarcoidosis and in the assessment of residual activity in patients with fibrotic pulmonary sarcoidosis [9]. Recently, PET-CT studies in sarcoidosis revealed that FDG uptake is variable in sarcoidosis and can mimic malignancies [10]. PET-CT findings in NSG are only reported in a few cases. PET-CT has a potential role in guidance of surgical biopsy and also demonstration of the extent of the disease [11]. In our case, the mass in the right middle lobe and nodules in the right lower lobe exhibited active FDG uptake, but mediastinal lymph nodes did not show any FDG uptake. Besides, there was no extrapulmonary involvement. In our opinion, albeit nonspecific in the diagnosis of NSG, PET-CT may demonstrate extrapulmonary involvement of the disease. More studies are needed to assess the utility of this method in distinguishing NSG from other pathologies.

Pathological features of NSG involve scattered nodules with a subpleural and peribronchovascular distribution and conglomerate masses with central cavitation [5]. Histologically, these nodules consist of confluent noncaseating granulomas, widespread zones of necrosis and vasculitis [12]. Granulomas are sarcoid-like and necrosis is in variable degrees [11]. These extensive areas of coagulative necrosis help to distinguish NSG from sarcoidosis [12]. Another differential diagnosis is Churg-strauss syndrome in which necrotizing granulomatous vasculitis is seen with eosinophilic infiltration [13]. Eosinophilia is also a characteristic of Wegener granulomatosis which is characterized by necrosis and granulomatous inflammation accompanied by a mixed cellular infiltrate [5]. Another entity in the differential diagnosis is bronchocentric granulomatosis which is characterized by a necrotizing granulomatous inflammation of bronchiolar epithelium with chronic inflammatory changes in the surrounding parenchyma [14]. Affected patients tend to have asthma, peripheral eosinophilia and positive sputum cultures for Aspergillus organism. Eosinophilia is not a characteristic of NSG, so histological evaluation helps in diagnosis, although radiological features of those diseases are similar.

The prognosis of NSG is usually benign and nodules or infiltrates may regress upon appropriate treatment or even without treatment [15].Treatment options are observation, medical therapy including steroids, or surgical resection for localized disease [7]. Rarely, NSG shows an aggressive course like sarcoidosis and immunosuppressive agents may be required. Vanishing lung syndrome is a rare manifestation of sarcoidosis or NSG. Miller discussed the relation between vanishing lung syndrome and NSG in 1981 [16]. After his comment, no cases have been reported on this task. In spite of a regular steroid therapy, vanishing lung syndrome developed in our case in a two-year period. Therefore, patients with NSG should be followed closely for the destructive manifestations and infectious agents such as tuberculosis.

In conclusion, although NSG shares features with sarcoidosis and other granulomatous angiitis, it is a separate entity. It has to be differentiated from other pathologies clinically, radiologically and histologically because of the differences in prognosis and response to treatment.

## References

[1] Liebow AA (1973). The J. Burns Amberson lecture--pulmonary angiitis and granulomatosis. Am Rev Respir Dis.

[2] Staples CA (1991). Pulmonary angiitis and granulomatosis. Radiol Clin North Am.

[3] Weisbrod GL (1989). Pulmonary angiitis and granulomatosis: a review. Can Assoc Radiol J.

[4] Quaden C, Tillie-Leblond I, Delobbe A, Delaunois L, Verstraeten A, Demedts M (2005). Necrotising sarcoid granulomatosis: clinical, functional, endoscopical and radiographical evaluations. Eur Respir J.

[5] Frazier AA, Rosado-de-Christenson ML, Galvin JR, Fleming MV (1998). Pulmonary angiitis and granulomatosis: radiologic-pathologic correlation. Radiographics.

[6] Dykhuizen RS, Smith CC, Kennedy MM, McLay KA, Cockburn JS, Kerr KM (1997). Necrotizing sarcoid granulomatosis with extrapulmonary involvement. Eur Respir J.

[7] Barreiro TJ, Gemmel DJ, Katzman PJ (2008). Necrotizing Sarcoid Granulomatosis. Clin Pulm Med.

[8] Feigin DS (1988). Vasculitis in the lung. J Thorac Imaging.

[9] Teirstein AS, Machac J, Almeida O, Lu P, Padilla ML, Iannuzzi MC (2007). Results of 188 whole-body fluorodeoxyglucose positron emission tomography scans in 137 patients with sarcoidosis. Chest.

[10] Prabhakar HB, Rabinowitz CB, Gibbons FK, O’Donnell WJ, Shepard JA, Aquino SL (2008). Imaging features of sarcoidosis on MDCT, FDG PET, and PET/CT. AJR Am J Roentgenol.

[11] Arfi J, Kerrou K, Traore S, Huchet V, Bolly A, Antoine M (2010). F-18 FDG PET/CT findings in pulmonary necrotizing sarcoid granulomatosis. Clin Nucl Med.

[12] Travis WD, Fleming MV (1996). Vasculitis of the lung. Pathology (Phila).

[13] Kim YK, Lee KS, Chung MP, Han J, Chong S, Chung MJ (2007). Pulmonary involvement in Churg-Strauss syndrome: an analysis of CT, clinical, and pathologic findings. Eur Radiol.

[14] Jeong YJ, Kim KI, Seo IJ, Lee CH, Lee KN, Kim KN (2007). Eosinophilic lung diseases: a clinical, radiologic, and pathologic overview. Ra diographics.

[15] Tauber E, Wojnarowski C, Horcher E, Dekan G, Frischer T (1999). Necrotizing sarcoid granulomatosis in a 14-yr-old female. Eur Respir J.

[16] Miller A (1981). The vanishing lung syndrome associated with pulmonary sarcoidosis. Br J Dis Chest.

